# Bessel-Hagen Disease: A Case Report of a Rare Disease

**DOI:** 10.7759/cureus.89651

**Published:** 2025-08-08

**Authors:** Fz Ammor, Mouad Gourti, Imane Lefqih, Elmehdi Maidi

**Affiliations:** 1 Thoracic Surgery, Centre Hospitalo-Universitaire (CHU) Souss Massa, Medical University of Agadir, Agadir, MAR

**Keywords:** chest wall, exostoses, non-malignant tumors, surgery, thoracic surgery

## Abstract

Bessel-Hagen disease, also known as hereditary multiple exostoses, is a rare genetic disorder characterized by the development of multiple benign bony outgrowths (osteochondromas), most commonly at the metaphyses of long bones. Although it typically presents in childhood, the clinical manifestations can vary greatly, ranging from asymptomatic masses to significant skeletal deformities. In this case report, we describe a young adult male with a family history of exostoses who presented with a painless, visible mass on his anterolateral chest wall, along with a similar lesion on the anterior tibia. Radiological evaluation confirmed multiple osteochondromas, without signs of malignancy or inflammation. Given the location and progressive nature of the chest lesion, surgical resection was performed for both functional and cosmetic reasons. The postoperative course was uneventful, and follow-up showed no recurrence. This case highlights the importance of individualized management in Bessel-Hagen disease, where surgical intervention may be beneficial even in the absence of pain or complications.

## Introduction

Bessel-Hagen disease is a rare disease with a genetic cause; alterations in several genes may be implicated. It is characterized by the presence of multiple intraosseous enchondromas located asymmetrically in the bones and whose location, sizes, and numbers vary considerably, ranging from the involvement of a single member to that of the entire skeleton. It often presents as multiple growths on the long bones, and standard imaging and CT scans do not confirm the diagnosis but may show signs of malignancy. Surgery is ideal, especially for complicated and symptomatic tumors.

## Case presentation

**Figure 1:**
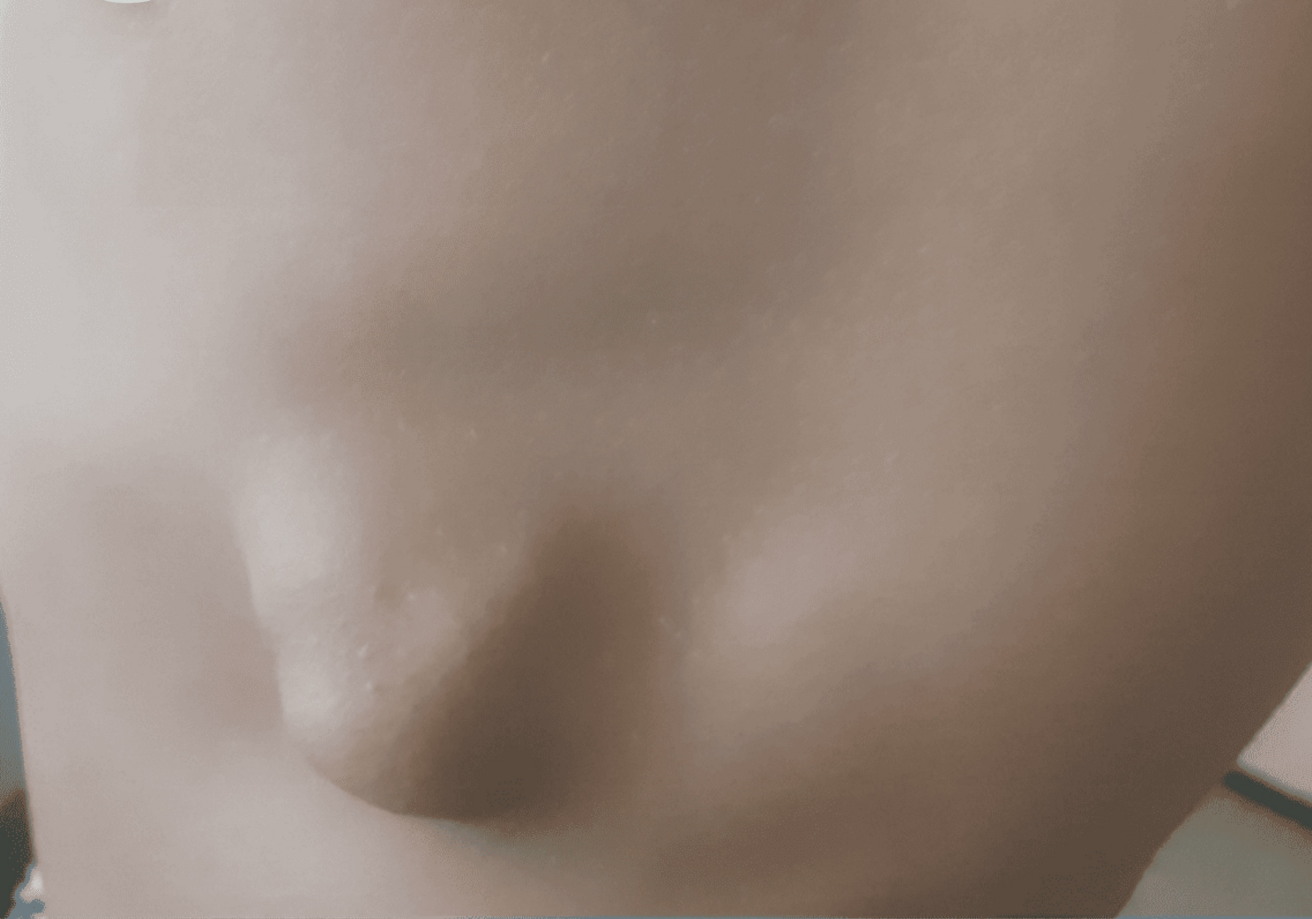
Anterolateral chest wall mass Clinical presentation of a firm, non-mobile swelling on the left anterolateral chest wall in an 18-year-old patient with hereditary multiple exostoses (Bessel-Hagen disease). The lesion was mildly tender on palpation, without overlying skin changes, inflammation, or signs of infection.

We report the case of an 18-year-old male who presented to our thoracic surgery clinic with chronic left-sided chest pain. His family history was notable for hereditary multiple exostoses, as his father had undergone three surgeries for limb osteochondromas. On physical examination, a firm, non-mobile, and tender swelling was palpated on the left anterolateral chest wall. There were no signs of inflammation or skin changes over the lesion. A second, non-painful bony prominence was observed on the anterior aspect of the left tibia, also without signs of inflammation. The patient was otherwise in good general health, with no signs of systemic illness or tumor invasion (Figure 1).

**Figure 2:**
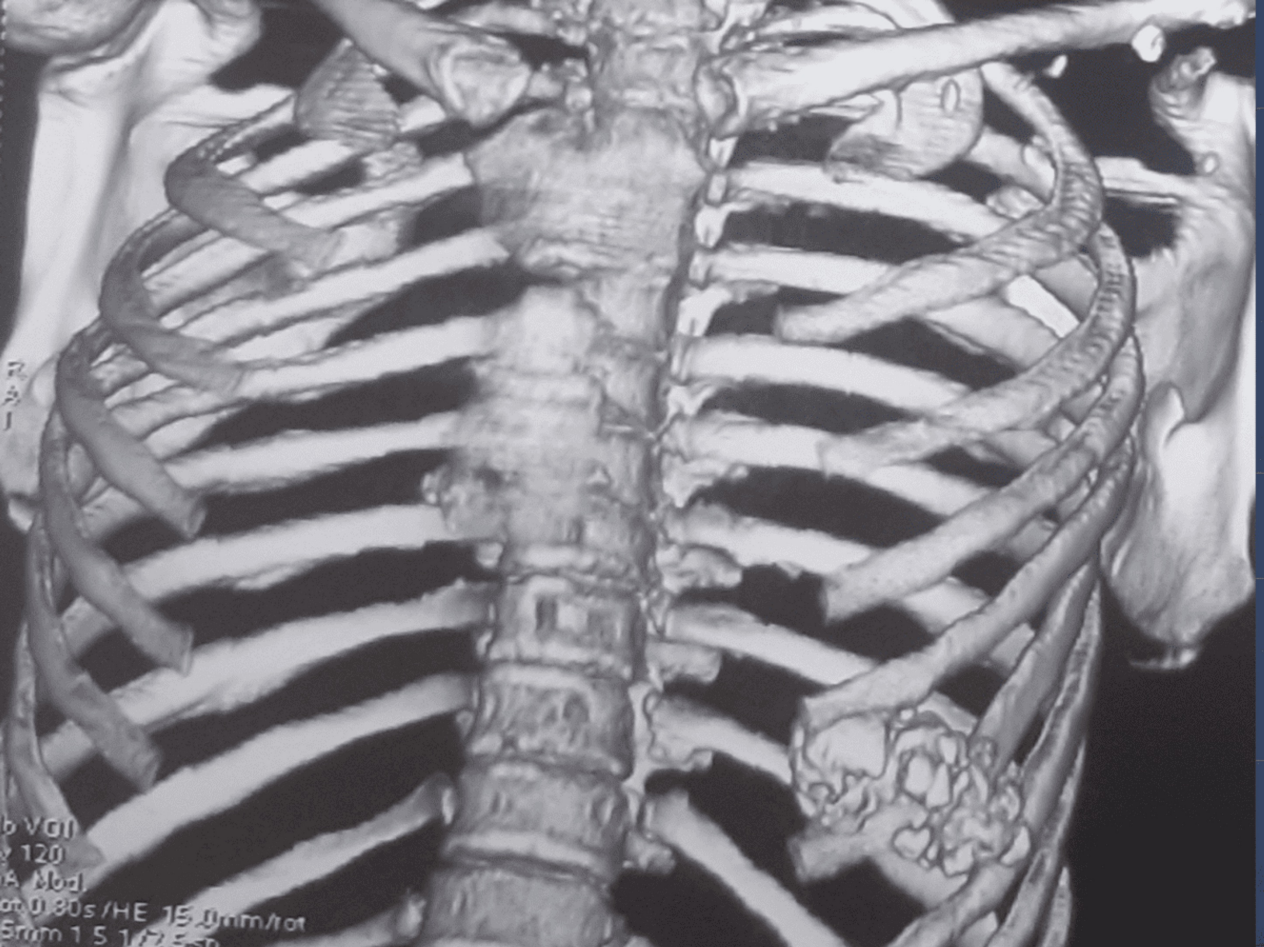
CT imaging of the chest wall showing the costal osteochondroma with no invasion of the pleura Three-dimensional reconstructed CT scan of the chest showing a costal osteochondroma arising from the left sixth rib. The lesion extends toward the intercostal spaces without evidence of pleural, pulmonary, or mediastinal invasion.

A standard chest radiograph revealed a well-defined bony outgrowth projecting from the sixth rib, extending into the adjacent intercostal spaces, without evidence of pleural or pericardial effusion. A thoracic CT scan confirmed a cortically and medullary continuous rib exostosis involving the sixth rib, extending both superiorly and inferiorly into neighboring intercostal spaces and medially toward the thoracic cavity. No signs of local invasion or compression of surrounding structures were noted (Figure 2).

Laboratory tests were within normal limits, including hemoglobin (13.2 g/dL), platelet count (300,000/mm³), renal and hepatic function, and coagulation profile (Table 1).

**Table 1:** Summary of laboratory test results

Test	Result	Reference Range	Interpretation
Hemoglobin	13.2 g/dL	13.0-17.0 g/dL	Normal
Platelet count	300,000/mm³	150,000-400,000/mm³	Normal
Renal function	Within normal limits	-	Normal
Hepatic function	Within normal limits	-	Normal
Coagulation profile	Within normal limits	-	Normal

**Figure 3:**
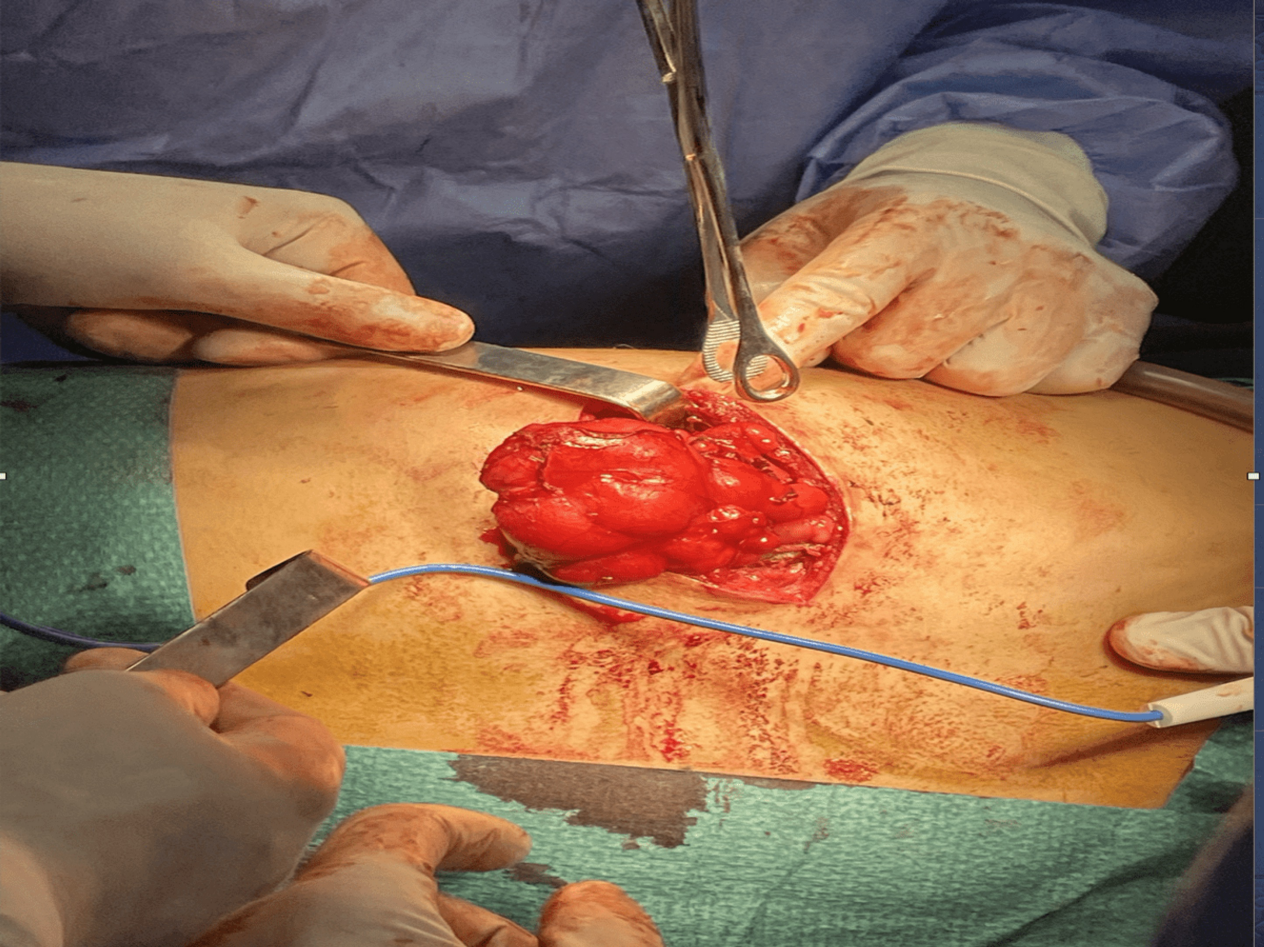
Intraoperative view of chest wall tumor excision Intraoperative image showing a large, well-circumscribed chest wall tumor being carefully dissected and mobilized. The mass appears vascularized and encapsulated. Multiple surgical instruments are used to maintain exposure and control the surrounding tissues. The procedure was performed under sterile conditions with appropriate hemostasis.

Given the persistent chest pain and potential risk of pleural or pericardial irritation, surgical resection was indicated. The patient was cleared for surgery and underwent excision under general anesthesia with selective right lung ventilation. He was positioned in the right lateral decubitus position. A 6-cm incision was made over the lesion. Intraoperatively, a firm, white mass was found extending into adjacent intercostal spaces and slightly projecting into the thoracic cavity, without invasion of the ribs or parietal pleura. The lesion was carefully dissected and completely removed. Local hemostasis was secured, and a medium-caliber pleural drain was placed. Wound closure was performed in layers, and an intercostal nerve block provided postoperative analgesia (Figure 3).

The postoperative course was uneventful. The pleural drain was removed on postoperative day 3, and the patient was discharged on day 5 with no complications. Follow-up confirmed good clinical recovery, with complete resolution of pain and no recurrence on imaging. Histopathological examination confirmed the diagnosis of a benign costal osteochondroma, with no signs of malignancy.

## Discussion

The prevalence of multiple exostoses disease (MEM) is estimated at 1/50,000 and appears to be more prevalent in males [1]. The number of osteochondromas can vary considerably both within and between families. The majority of cases are asymptomatic and occur in bones that develop from cartilage, especially the long bones of the extremities, primarily around the knee. Bone lesions on flat bones, vertebrae, and ribs are less common, and the head is usually not affected [1]. Several studies have incriminated mutations in two genes: exostosin-1 (EXT1) and exostosin-2 (EXT2) [1-3]. Most often asymptomatic, but pain and functional discomfort are the most common signs that lead to a request for care and may represent signs of complications or malignant transformations [2]. Reduced joint movement, valgus or varus deformity, angulation, and a pathological fracture may be discovery signs of the disease, which is often clinically reduced and underestimated [3,4]. Malignant transformation of osteochondromas is a rare but important complication, mostly observed in adult patients [4,5]. Multiple exostoses are diagnosed clinically and radiologically, and imaging can often eliminate signs of malignancy by showing a well-limited excrescent mass that respects the cortex and medulla of the affected bone without locoregional invasion or border irregularities, demineralization, or inhomogeneous calcifications without any osteolytic areas [2,4,5]. There is no medical treatment that modifies the disease, but in the absence of clinical problems, osteochondromas do not require therapy. Surgical resection is often essential when osteochondromas cause pain, interfere with joint or muscle function, compress nerves or vessels, or cause deformity [2,6,7]. Costal wall disease in multiple exostoses is rare, and its management depends on the histologic type. Most chest wall tumors are treated with surgical resection and reconstruction as the first line of therapy. Reconstruction often uses a combination of myocutaneous flaps and prosthetic materials. The presence of a malignant pleural effusion is a contra-indication to surgical resection [7,8].

## Conclusions

Multiple exostoses is a rare disease with a clinical-radiological diagnosis. Osteochondromas are characterized by continuity of the cortical and medullary bone with respect to normal bone. The risk of malignant degeneration has been described in the literature, and surgical removal is the ideal treatment, especially in symptomatic or complicated forms.
